# Unusual Case of Giant Nonthrombosed Right Coronary Artery Pseudoaneurysm With Coronary Artery Fistula

**DOI:** 10.1016/j.jaccas.2021.01.015

**Published:** 2021-03-10

**Authors:** Yoshito Ito, Satoshi Kainuma, Yasushi Yoshikawa, Koichi Toda, Shigeru Miyagawa, Hiroki Hata, Daisuke Yoshioka, Seiko Ide, Yoshiki Sawa

**Affiliations:** a Department of Cardiovascular Surgery, Osaka University Graduate School of Medicine, Suita, Osaka, Japan; b Department of Cardiology, Osaka University Graduate School of Medicine, Suita, Osaka, Japan

**Keywords:** coronary artery aneurysm, coronary artery pseudoaneurysm, coronary artery fistula, right ventricular tumor, ruptured coronary aneurysm, three-dimensional computed tomography, CAF, coronary artery fistula, CT, computed tomography, RCA, right coronary artery, RV, right ventricle, SVG, saphenous vein graft

## Abstract

Coronary artery aneurysm and pseudoaneurysm are rare and mainly result from atherosclerosis. We present a successfully treated case of a giant right coronary artery aneurysm and pseudoaneurysm with a coronary artery fistula, which might have developed after cardiac surgery for a right ventricular tumor 35 years earlier. (**Level of Difficulty: Advanced.**)

## Central Illustration

**Figure:**
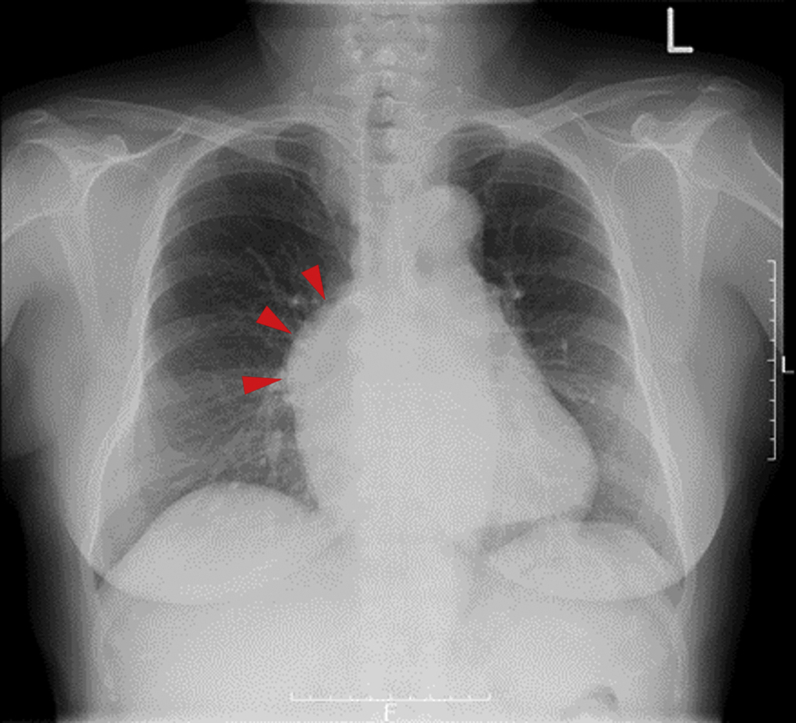

## History of Presentation

A 63-year-old woman presented with a sudden onset of chest tightness and attended a primary hospital 12 h after the onset. Her heart rate was 53 beats/min, her blood pressure was 118/57 mm Hg, and her oxygen saturation was 98% on room air. There was no difference in blood pressure between the limbs, and no abnormal heart murmurs were detected. Electrocardiography showed an ST-segment elevation in the inferior leads, with an elevated troponin I value of 420 pg/ml and normal levels of creatine phosphokinase. She did not develop heart failure symptoms such as shortness of breath and leg edema.

**Learning Objectives**

• To understand the anatomical relationships between RCA aneurysm, pseudoaneurysm, and RCA fistula in CT.
• To consider a previous cardiac surgery as a potential cause of coronary artery aneurysm and fistula.

## Past Medical History

The patient had undergone right ventricular tumor resection via median sternotomy 35 years earlier, with no major complications thereafter. The histological examination had revealed a hemangioendothelioma. She had no other risk factors of atherosclerosis except for hypertension.

## Differential Diagnosis

Based on the patient’s clinical symptoms and the findings of electrocardiography, the differential diagnosis included angina, acute myocardial infarction, acute aortic dissection, and pulmonary thromboembolism.

## Investigations

**Figure 1:**
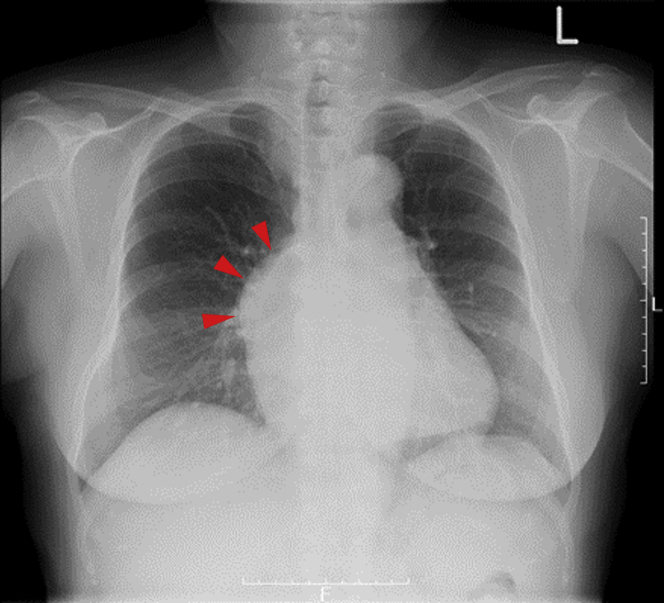
Pre-Operative Chest Radiography Abnormal shadow on the right side of the heart **(red arrowheads)**.

**Figure 2:**
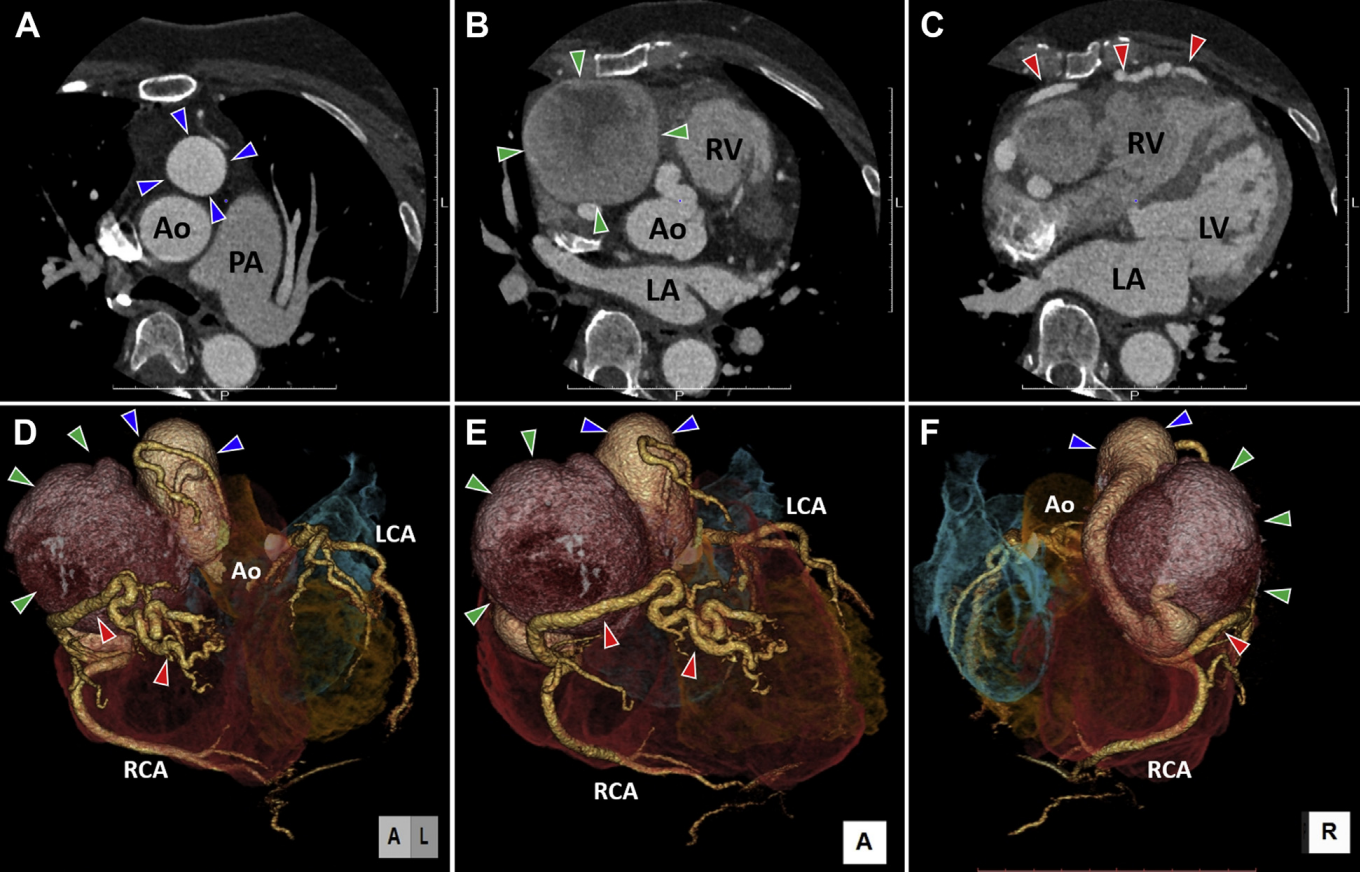
Pre-Operative Enhanced Computed Tomography Imaging **(A)** The right coronary aneurysm (diameter 28 mm; **blue arrowheads**) anterior to the aorta (Ao) (axial image). **(B)** Giant pseudoaneurysm (maximum size 63 mm; **green arrowheads**) on the right side of the heart. **(C)** Abnormally dilated fistulas **(red arrowheads)** branching off the right coronary artery (RCA) and communicating with the right ventricle (RV). **(D to F)** 3-dimensional computed tomography imaging showing the anatomical relation between the pseudoaneurysm **(green arrowheads)**, right coronary aneurysm **(blue arrowheads)**, and coronary artery fistula **(red arrowheads)**. RCA just distal to the coronary artery fistula is healthy. **D** is the left anterior view; **E** is the anterior view; and **F** is the right view. Note that inflows of the pseudoaneurysm, coronary fistula, and healthy RCA were branched off from the right coronary aneurysm. LA = left atrium; LCA = left coronary artery; LV = left ventricle; PA = pulmonary artery.

Chest radiography showed an abnormal shadow on the right side of the heart (Figure 1). Echocardiography showed an abnormal flow into the right ventricle (RV) and an abnormal mass shadow without asynergy anterior to the RV. To gain further information, enhanced computed tomography (CT) imaging was performed before coronary angiography. Enhanced CT imaging showed a right coronary artery (RCA) aneurysm with a diameter of 28 mm, anterior to, and on the left of, the ascending aorta (Figures 2A and 2B). In addition, a giant saccular RCA aneurysm (maximum size 63 mm) with a different CT value was located between the sternum and ascending aorta on the right and lay on the right atrioventricular groove (Figures 2B and 2C). Furthermore, abnormally dilated fistulas were seen horizontally located anterior to the giant aneurysm and RV (Figure 2C).

On three-dimensional CT imaging, the left coronary artery system was intact, and the fistula branched off the RCA distal to the aneurysm and communicated with the RV (Figures 2D and 2E). The giant saccular RCA aneurysm located distal to the proximal RCA aneurysm seemed to be pressing on the proximal RCA posteriorly (Figures 2D to 2F, Video 1). Oximetry revealed an oxygen step-up between the right ventricular outflow (80.2%) and middle right atrium (69.5%), and an estimated left-to-right shunt of 17.4%, which were consistent with coronary artery fistula (CAF) communication with the RV.

## Management

**Figure 3:**
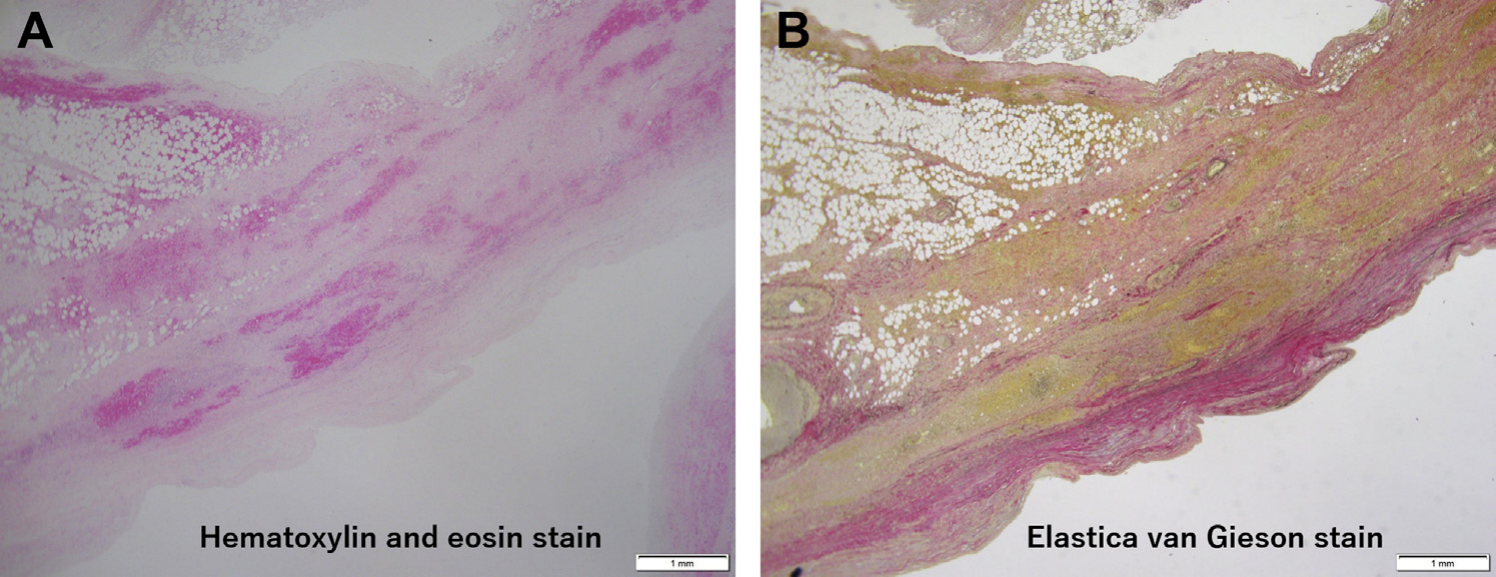
Pathological Findings of the Saccular Aneurysm Lack of vessel wall integrity. **(A)** Hematoxylin and eosin stain. **(B)** Elastica van Gieson stain.

A deﬁnitive repair procedure was scheduled. During the operation, tight adhesions between the sternum and the aneurysm were carefully dissected. A saphenous vein graft (SVG) was anastomosed to the RCA distal to the fistula. After institution of a standard cardiopulmonary bypass with aortic cross-clamping, an antegrade cardioplegic infusion was administered. The giant saccular aneurysm seemed to be a pseudoaneurysm because of the extremely thin walls. The antegrade infusion into the aortic root conﬁrmed a single hole located posteriorly in the aneurysm. The proximal aneurysm was dissected from the RV and resected as much as possible. The aneurysm was considered a true aneurysm based on the macroscopic appearance of the aneurysmal wall. The RCA orifice, which was 12 mm in diameter, was closed with bovine pericardium. The CAF was then ligated and transected. The proximal anastomosis to the SVG was then completed, and the proximal portion of the distal anastomosis to the SVG was ligated. The aortic cross-clamp was released, and the cardiopulmonary bypass was weaned successfully (Supplemental Video 2A, Supplemental Video 2B, Supplemental Video 2C). The giant saccular aneurysm was histologically confirmed as a pseudoaneurysm following the lack of vessel wall integrity (Figure 3).

## Discussion

This case was a combination of rare pathologies: a giant nonthrombosed coronary artery aneurysm, pseudoaneurysm, and CAF. Giant coronary artery aneurysms are rare and defined by a diameter ≥2 cm; they occur in ∼0.15% to 4.9% of patients who undergo coronary angiography (1,2). CAF is also a rare abnormal vessel communication between the coronary artery and a cardiac chamber or great vessel, with a prevalence of 0.1% (3). In addition to the impressive size and images of the lesion, this case included a few other novel findings; one was the pathogenesis of these complications. Common causes of RCA pseudoaneurysm in adults are atherosclerosis, followed by trauma, Behçet’s disease, and CAF (Supplemental Table 1); CAF was the only underlying disease in our patient. Moreover, the RCA aneurysm was neither atherosclerotic nor calcified. No signs of atherosclerosis were found, and the vascular system except the RCA was normal on CT imaging, suggesting that the patient had a low risk for coronary events.

Because the aneurysm extended from the right coronary sinus to just proximal to the CAF, and the RCA immediately distal to the CAF was healthy, we speculated that the aneurysm developed secondary to CAF, presumably owing to the high blood flow from the RCA to the RV through the CAF. In addition, the patient’s coronary system was normal, and no fistulas were detected during her first cardiac surgery (i.e., right ventricular tumor resection). Therefore, the CAF in this patient was acquired, and not congenital; unlike most CAFs, which are congenital, it probably developed in association with the first cardiac surgery. This also suggests that the aneurysms of the RCA gradually developed over the last 30 years. Coronary pseudoaneurysm concomitant with true aneurysm is also relatively uncommon (Supplemental Table 1). To the best of our knowledge, this report is the first on coronary aneurysm, pseudoaneurysm, and CAF that developed in association with a previous right ventricular tumor surgery.

Previous studies have reported that coronary aneurysms are mostly associated with thrombus formation (2,4) (Supplemental Table 1). It was therefore reasonable to hypothesize that the myocardial infarction was caused by a thrombus from the RCA aneurysm. However, no thrombus was seen in the coronary aneurysm and/or pseudoaneurysm on pre-operative CT scanning or intraoperatively. This could be because the high blood flow in the aneurysm secondary to the high-pressure gradient between the RCA and RV prevented thrombus formation. Instead, this suggested that the myocardial infarction was not due to thromboembolism but due to the rupture of the proximal RCA aneurysm, which might have resulted in the sudden loss of a large amount of blood, thereby leading to persistent coronary ischemia. This theory can be supported by the fact that no abnormalities were found on radiography until the coronary event occurred. The tight adhesion in the pericardial space might have prevented massive bleeding of the ruptured coronary aneurysm, and consequent cardiac tamponade. Although the optimal management strategy has not been established because of the rarity of this disorder, we believe that the indication for surgery was appropriate for this patient. Our case provided a new insight into the development of CAF and RCA aneurysm, probably from a prior right ventricular resection surgery.

## Follow-Up

**Figure 4:**
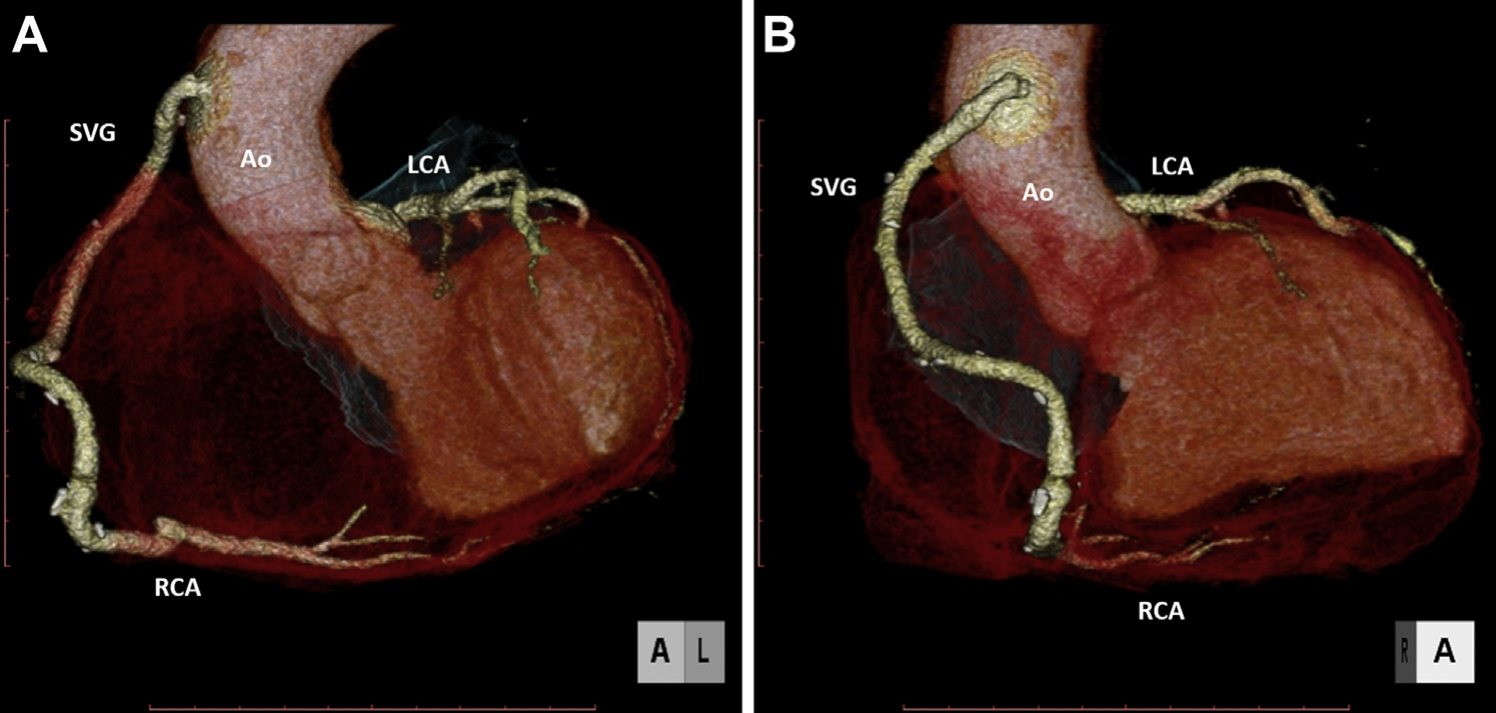
Post-Operative Enhanced Computed Tomography Imaging Pseudoaneurysm, right coronary aneurysm, and abnormal coronary fistula were completely resected. **(A)** Left anterior view. **(B)** Anterior view. SVG = saphenous vein graft; other abbreviations as in Figure 2.

No major post-operative adverse cardiac events were observed. Post-operative CT imaging showed a patent vein graft and successful resection of the aneurysms, with no abnormal fistula (Figure 4, Video 3). The echocardiogram showed a good cardiac function. The patient was discharged on post-operative day 29.

## Conclusions

We presented a rare case of giant coronary artery aneurysm, pseudoaneurysm, and CAF, which might have originated from a previous right ventricular tumor resection. Patients undergoing right ventricular tumor resection should be meticulously followed up, as coronary aneurysm and fistula can develop postoperatively and may lead to subsequent coronary events.

## Funding Support and Author Disclosures

The authors have reported that they have no relationships relevant to the contents of this paper to disclose.

## Appendix

Supplemental Video 1 Pre-Operative CT Imaging Pre-operative computed tomography (CT) imaging of the anatomical relations between the right coronary aneurysm, pseudoaneurysm, and right coronary fistula.

Supplemental Video 2A Operation Procedure(A) First, we anastomosed the vein graft to the healthy right coronary artery (RCA) just distal to the end of the right coronary aneurysm. We then opened the saccular aneurysm and detected the inflow hole located posteriorly in the aneurysm. The saccular aneurysm was considered a pseudoaneurysm macroscopically because of the thin walls. We also opened the proximal aneurysm, and resected the vessel communication between the two coronary aneurysms as much as possible. **(B)** The RCA orifice was closed by using a bovine pericardium patch. We then transected the fistulas communicating with the right ventricle. The right coronary was ligated just proximal to the vein graft anastomosis. **(C)** Finally, we anastomosed the vein graft to the ascending aorta and completed the bypass from the ascending aorta to the RCA.

Supplemental Video 2B Operation Procedure(A) First, we anastomosed the vein graft to the healthy right coronary artery (RCA) just distal to the end of the right coronary aneurysm. We then opened the saccular aneurysm and detected the inflow hole located posteriorly in the aneurysm. The saccular aneurysm was considered a pseudoaneurysm macroscopically because of the thin walls. We also opened the proximal aneurysm, and resected the vessel communication between the two coronary aneurysms as much as possible. **(B)** The RCA orifice was closed by using a bovine pericardium patch. We then transected the fistulas communicating with the right ventricle. The right coronary was ligated just proximal to the vein graft anastomosis. **(C)** Finally, we anastomosed the vein graft to the ascending aorta and completed the bypass from the ascending aorta to the RCA.

Supplemental Video 2C Operation Procedure(A) First, we anastomosed the vein graft to the healthy right coronary artery (RCA) just distal to the end of the right coronary aneurysm. We then opened the saccular aneurysm and detected the inflow hole located posteriorly in the aneurysm. The saccular aneurysm was considered a pseudoaneurysm macroscopically because of the thin walls. We also opened the proximal aneurysm, and resected the vessel communication between the two coronary aneurysms as much as possible. **(B)** The RCA orifice was closed by using a bovine pericardium patch. We then transected the fistulas communicating with the right ventricle. The right coronary was ligated just proximal to the vein graft anastomosis. **(C)** Finally, we anastomosed the vein graft to the ascending aorta and completed the bypass from the ascending aorta to the RCA.

Supplemental Video 3 Post-Operative CT Imaging Post-operative computed tomography (CT) imaging of the patent saphenous vein graft and the successful resection of the right coronary artery aneurysms, with no abnormal fistula residue.

## References

[1] Kimura S., Miyamoto K., Ueno Y. (2006). Cardiac tamponade due to spontaneous rupture of large coronary artery aneurysm. Asian Cardiovasc Thorac Ann.

[2] Li D., Wu Q., Sun L. (2005). Surgical treatment of giant coronary artery aneurysm. J Thorac Cardiovasc Surg.

[3] Gowda R.M., Vasavada B.C., Khan I.A. (2006). Coronary artery fistulas: clinical and therapeutic considerations. Int J Cardiol.

[4] Keyser A., Hilker M.K., Husser O., Diez C., Schmid C. (2012). Giant coronary aneurysms exceeding 5 cm in size. Interact Cardiovasc Thorac Surg.

