# Supplementary material for: Unusual Case of Giant Nonthrombosed Right Coronary Artery Pseudoaneurysm With Coronary Artery Fistula
Source: JACC Case Rep. 2021 Mar 10;3(5):806–10. doi: 10.1016/j.jaccas.2021.01.015 (PMC8311150; doi:10.1016/j.jaccas.2021.01.015)
Supplement: Supplemental Table 1 [file mmc6.pdf]

Supplemental Appendix

Supplemental Table. Previous reports of RCA pseudoaneurysm

Author	Age	Sex	Etiology	Presentations	Past procedure history	Thrombus in pseudo-aneurysm	Coexisting RCA true aneurysm	Treatment
Frischknecht et al. (5)	55	M	Atherosclerosis	Angina, Heart failure	-	N/A	+	Surgery
Honda et al. (6)	61	M	Atherosclerosis	Angina	-	+	-	Surgery
Cabarrus et al. (7)	59	M	Atherosclerosis	Heart failure	PCI	+	-	Surgery
Pontone et al. (8)	65	M	Atherosclerosis	Angina	PCI	+	-	Surgery
Tos et al. (9)	56	M	Atherosclerosis	Angina	PCI	+	-	Surgery
Pandey et al. (10)	49	M	Atherosclerosis	Angina, Heart failure	PCI	+	-	Surgery
Lell et al. (11)	73	M	Atherosclerosis	Asymptomatic	PCI	N/A	-	PCI
Wu et al. (12)	62	F	Atherosclerosis	Heart failure	PCI	+	-	Surgery
Caruso et al. (13)	50	M	Atherosclerosis	Angina	PCI	N/A	-	PCI
Liu et al. (14)	42	M	Trauma	Angina	-	+	-	Surgery
Yoshioka et al. (15)	69	M	Trauma	Asymptomatic	-	+	-	Surgery
Iemura et al. (16)	51	F	Trauma	Asymptomatic	-	+	-	Surgery
Kasapis et al. (17)	30	F	Bechet's disease	Angina	-	+	-	PCI
Kaseda et al. (18)	35	F	Bechet's disease	Angina	-	+	-	Surgery

Berry et al. (19)	31	M	Angiosarcoma	Angina	-	+	-	Surgery
Stessman-Lande et al. (20)	83	N/A	N/A	Shock	-	+	-	PCI
Darbar et al. (21)	37	F	N/A	Angina	-	+	+	Surgery
Izutani et al. (22)	67	M	N/A	Angina	-	+	+	Surgery
Wang et al. (23)	35	F	N/A	Heart failure	-	+	-	Surgery
Dai et al. (24)	69	F	N/A	Angina	-	+	-	PCI
Yang et al. (25)	28	M	N/A	Palpitations	-	+	-	Surgery
Bhagwat et al. (26)	38	F	Coronary fistula (congenital)	Heart failure	-	+	-	Surgery
Ito et al. (present case)	63	F	Coronary fistula (acquired)	Angina, Myocardial infarction	RV tumor resection	-	+	Surgery

The table summarizes 23 case reports of RCA pseudoaneurysm including our case. The average age is 52.5 years old. The number of male and female patients is 13 and 9, respectively. On admission, fourteen patients had angina, six had symptoms of heart failure like shortness of breath, and two had palpitations. One patient presented with shock due to cardiac tamponade. Three patients were asymptomatic. About the etiologies, seven patients underwent PCI, three patients experienced trauma, nine patients had atherosclerosis, two patients had Bechet's syndrome, and one patient had angiosarcoma. The etiologies of the other six patients are unclear; probably, they had congenital disorders of the coronary artery. Two patients had a pseudoaneurysm complicated with a coronary fistula (our patient included), and only our patient had an 'acquired' fistula. Additionally, only our patient underwent past cardiac surgery. Almost all patients except ours had pseudoaneurysms associated with a thrombus. Pseudoaneurysms complicated by RCA aneurysms were observed in four cases (our case included). As

for treatment, 18 cases underwent surgical treatment, while five cases underwent PCI. Four deaths were reported.

Abbreviations: PCI = percutaneous coronary intervention, N/A = not assessed.

References of supplemental table

5) Frischknecht JK, Shander D, Kurt TL, Wolf PS, Craddock LD. Spontaneous Rupture of a Coronary Artery with False Aneurysm Formation – Successful Surgical Repair. Chest. 1977; 72: 123 – 125.

6) Honda T, Kawano H, Tsuneto A et al. Coronary Artery Pseudoaneurysm due to Medial Mucoid Degeneration Mimicking an Intra-atrial Mass. Intern Med. 2015; 54: 2453 – 2458.

7) Cabarrus M, Yang B, Schiller N, Miller C, Ordovas K. Iatrogenic Giant Artery Pseudoaneurysm With “Daughter Aneurysm” Formation. J Thorac Imaging. 2012; 27: W185 – 187.

8) Pontone G, Cavallotti L, Bertella E, Andreini D, Lualdi A, Alamanni F. An Unusual Presentation of Giant Right Coronary Artery Pseudoaneurysm as a Late Complication of Stent Fracture Treated by Hybrid Procedure. JACC Cardiovasc Interv. 2014; 7: e145 – 146.

9) Los A, Nowak R, Mielczarek M, Kozaryn R, Jagielak D, Siondalski P. Right atrium tumor – pseudoaneurysm of right coronary artery. A rare complication after percutaneous coronary intervention. Adv Interv Cardiol. 2017; 13, 4: 341 – 342.

10) Nirmal N, Sharma A, Kumar S. right coronary artery pseudoaneurysm post everolimus eluting stent implantation causing tamponade. BMJ Case Rep. 2018; 2018: bcr2018227714.

11) Lell E, Wehr G, Sechtem U. Delayed Development of a Coronary Artery Pseudoaneurysm After Angioplasty. Cathet Cardiovasc Intervent. 1999; 47: 186 – 190.

12) Wu IH, Koullias GJ, Dewar ML, Henry GA. Hemodynamic Compromise From a Right Coronary Artery Pseudoaneurysm After Remote Stent Placement. Ann Thorac Surg. 2005; 79: 1062.

13) Caruso M, Evola S, Fattouch K et al. Angina Due to Late Huge Coronary Pseudoaneurysm Following Stent Implantation. Intern Med. 2011; 50: 577 – 579.

14) Liu L, Li Z, He Y, Gu X, Nixon JV. Coronary Artery Pseudoaneurysm Following Blunt Trauma. J Card Surg. 2012; 27: 563 – 565.

15) Yoshioka D, Izutani H, Gyugo M, Kawachi K, Sawa Y. Asymptomatic Giant Traumatic Right Coronary Artery Pseudoaneurysm Caused by Sternal Fracture. Ann Thorac Surg. 2011; 92: e33 – 35.

16) Iemura J, Oku H, Shirotani H. Right coronary artery pseudoaneurysm after blunt injury to the chest. Heart. 1996; 76: 86.

17) Kasapis C, Grossman PM, Chetcuti SJ. Percutaneous treatment of a giant right coronary artery pseudoaneurysm in Adamantiades – Behcet’s syndrome. Eur Heart J. 2009; 30: 2554 – 2555.

18) Kaseda S, Koiwaya Y, Tajimi T et al. Huge false aneurysm due to rupture of the right coronary artery in Behcet’s syndrome. Am Heart J. 1982; 103: 569 – 571.

19) Berry MF, Williams M, Welsby I, Lin S. Cardiac Angiosarcoma Presenting With Right Coronary Artery Pseudoaneurysm. J Cardiothrac Vasc Anesth. 2010; 24: 633 – 635.

20) Stressman - Lande I, Salem R, Rubinstein C, Hiller N, Heyman SN, Alcalai R. Cardiac tamponade and coronary artery pseudoaneurysm after brachial

arterial embolectomy, possible role for an aberrant origin of the right coronary artery. J Vasc Surg Cases and Innovative Techniques. 2018; 4: 27 - 30.

21) Darbar D, Patel PJ, Devine SM, Schaff HV, Click R. Congenital Right Coronary Artery Aneurysm Causing Myocardial Infarction, Pseudoaneurysm Formation, and Right Atrial Compression. J Am Soc Echocardiogr. 2002; 15: 736 – 738.

22) Izutani H, Shibukawa T, Kawamoto J, Ishibashi K. Spontaneous right coronary artery pseudoaneurysm. Gen Thorac Cardiovasc Surg. 2007; 55: 259 – 261.

23) Wang YC, Hsu RB, Huang CH. Spontaneous giant right coronary artery pseudoaneurysm. J Thorac Cardiovasc Surg. 2014; 148: 349 – 350.

24) Dai HL, Guang XF, Jiang LH, Xue Q, Zhang WH. Overlapping – Stent Intervention Treatment of a Giant Right Coronary Artery Pseudoaneurysm. JACC Cardiovasc Interv. 2015; 8: e255 – 256.

25) Yang K, Zhao S. Giant Pseudoaneurysm Caused by Ruptured Coronary Artery Aneurysm Presenting as a Right Paracardial Mass. Cir Cardiovasc Imaging. 2019; 12: e008788. [E-pub ahead of print]

26) Bhagwat K, Jaria R, Shetty V, Gandhe U, Pandey K. Giant Calcified Pseudoaneurysm of Right Coronary Artery Presenting as a Right Intra-Atrial Mass. Ann Thorac Surg. 2010; 89: 969 – 971.
